# Does changing perceptions of sleep by incorporating sleep wearables improve insomnia? Protocol for a randomized study (the Novel Insomnia Treatment Experiment)

**DOI:** 10.1093/sleepadvances/zpad012

**Published:** 2023-02-16

**Authors:** Marie-Antoinette Spina, Thomas Andrillon, Joshua F Wiley, Shantha M W Rajaratnam, Bei Bei

**Affiliations:** Turner Institute for Brain and Mental Health, School of Psychological Sciences, Faculty of Medicine, Nursing and Health Sciences, Monash University, Clayton, VIC, Australia; School of School of Philosophical, Historical, and International Studies, Centre for Consciousness and Contemplative Studies, Monash University, Melbourne, VIC, Australia; Paris Brain Institute, Sorbonne Université, Inserm-CNRS, Paris, France; Turner Institute for Brain and Mental Health, School of Psychological Sciences, Faculty of Medicine, Nursing and Health Sciences, Monash University, Clayton, VIC, Australia; Turner Institute for Brain and Mental Health, School of Psychological Sciences, Faculty of Medicine, Nursing and Health Sciences, Monash University, Clayton, VIC, Australia; Turner Institute for Brain and Mental Health, School of Psychological Sciences, Faculty of Medicine, Nursing and Health Sciences, Monash University, Clayton, VIC, Australia; Women’s Mental Health Service, Royal Women’s Hospital, Parkville, VIC, Australia

**Keywords:** Sleep, Sleep–wake estimation, Sleep–wake state misperception, Insomnia disorder, Dreem, Fitbit

## Abstract

**Study Objectives:**

Insomnia is common in the general population and is diagnosed based on self-reported sleep complaints. There is a frequent discrepancy between objectively recorded and self-reported sleep (sleep–wake state discrepancy), especially in individuals with insomnia. Although sleep–wake state discrepancy is well-documented in the literature, it is not well understood. This protocol describes the methodology of a randomized control study, which will examine whether providing monitoring and feedback about objectively recorded sleep with support for interpretation of sleep–wake state discrepancy improves insomnia symptoms and will explore the potential mechanisms of change.

**Methods:**

Participants are 90 individuals with insomnia symptoms (Insomnia Severity Index [ISI] ≥10). Participants will be randomized to one of two conditions: (1) Intervention: feedback about objectively recorded sleep (actigraph and optional electroencephalogram headband) with guidance for data interpretation, (2) Control: sleep hygiene session. Both conditions will involve individual sessions and two check-in calls. The primary outcome is ISI score. Secondary outcomes include sleep-related impairment, symptoms of anxiety and depression, and other sleep and quality of life measures. Outcomes will be assessed using validated instruments at baseline and post-intervention.

**Discussion:**

With increasing number of wearable devices that measure sleep, there is a need to understand how sleep data provided by these devices could be utilized in the treatment of insomnia. Findings from this study have the potential to better understand sleep–wake state discrepancy in insomnia and uncover new approaches to supplement current insomnia treatment.

**Statement of Significance**

Numerous wearable devices provide sleep data, but few provide feedback tailored to individuals with insomnia. Current insomnia treatments do not routinely utilize data from wearable devices. This adequately powered, randomized study could fill this gap by investigating whether providing monitoring and feedback about recorded sleep, with support for interpretation of sleep–wake state discrepancy, could improve insomnia symptoms. Findings have the potential to better understand sleep-state perception in insomnia and uncover new approaches to supplement current insomnia treatment.

## Introduction

Insomnia is characterized by dissatisfaction with sleep, including difficulty initiating or maintaining sleep, and/or early-morning awakenings [1], with associated daytime impairment such as fatigue and mood disturbances [2]. 9% to 30% of individuals in the general population experience symptoms of insomnia [3–6]. The diagnosis of insomnia disorder is based on self-reported sleep complaints, without the need for recorded sleep data [1, 7]. Individuals with insomnia often underreport total sleep time (TST), and overreport sleep onset latency (SOL; time to fall asleep) and wake after sleep onset [8–11] compared to recorded sleep. The difference between self-reported and objectively measured sleep parameters is known in the literature as sleep-state misperception [12–14], paradoxical insomnia [15–17], or sleep discrepancy [18–20]. In this manuscript, we will use the term sleep–wake state discrepancy [21] to define differences that occur when comparing objectively recorded versus self-reported sleep data.

Sleep–wake state discrepancy is a common phenomenon observed in 9%–50% of individuals with insomnia [22–26]; it has also been observed in other sleep disorders including sleep apnea [27–29], periodic limb movement disorder [26], narcolepsy [30], as well as adults in the general community [31–33]. Although sleep–wake state discrepancy may include longer or better perceived (vs. objectively measured) sleep (e.g. “reverse sleep state misperception,” [34] “positive sleep state misperception” [35]), shorter and poorer perceived sleep has been consistently linked to greater insomnia symptoms. For example, individuals with insomnia and higher sleep–wake state discrepancy self-report poorer sleep (i.e. lower sleep quality, shorter TST, longer SOL, and lower sleep efficiency) and higher insomnia symptom severity compared to those with lower sleep–wake state discrepancy [20].

The underlying mechanisms of sleep–wake state discrepancy are not well understood, but the potential perspectives can be grouped into the following categories: physiological aspects include local modulations of sleep depth (i.e. local sleep) and sensitivity to sleep fragmentation [36–41]. Measurement-related aspects include differences in how tools assess sleep, as each tool infers sleep in a different way (e.g. polysomnography from electroencephalogram signals, actigraphy from activity and movement) [21]. Cognitive-related aspects include sleep-related worries and selective monitoring [42, 43]. Harvey’s cognitive model of insomnia suggests that concerns and worries about sleep and associated daytime consequences could lead to increasing sleep monitoring, which can result in overestimating sleep difficulties [42]. Therefore, sleep-related thoughts and beliefs appear to play an important role in the perception of sleep and the development of insomnia.

Sleep–wake state discrepancy has been shown to be modifiable [16, 44, 45], and reducing it may improve sleep complaints. Downey et al. [46] provided individuals with insomnia feedback about their sleep via polysomnography (PSG) by waking them up 27 times per night. After feedback, participants more accurately estimated their sleep/wake state and reported an improved ability to fall asleep. Tang et al. [44] conducted a pilot study (*N* = 40), in which individuals with insomnia were randomly allocated into either “shown discrepancy” or “non-demonstration” groups. The “shown-discrepancy” group was taught to read actigraph data and calculate sleep–wake state discrepancy; subsequently, they reported shortened SOL and less anxiety and preoccupation about sleep compared to the “non-demonstration” group [44]. Tang et al. [45] expanded on those findings with another experiment (*N* = 48), in which the control condition was verbally told about sleep–wake state discrepancy without demonstration or calculation. Consistent with findings from the earlier study, those who completed the intervention had a greater reduction in self-reported sleep impairment, insomnia symptoms, and sleep-related anxiety and distress [45].

On the contrary, receiving “negative feedback” (e.g., being told that last night’s sleep was poor quality) about sleep may have adverse effects. For example, one study (*N* = 22) found that providing individuals with insomnia “negative” feedback about their sleep was associated with increased negative thoughts, sleepiness, monitoring for sleep-related threats, and daytime safety behaviors, relative to “positive” sleep feedback [47]. Similarly, a more recent study (*N* = 63) found that giving individuals with insomnia “negative” feedback about their sleep resulted in significantly lower self-reported alertness and significantly higher levels of self-reported sleepiness and fatigue compared to those given “positive” sleep feedback [48].

Cognitive behavioral therapy for insomnia (CBT-I), the first-line treatment for chronic insomnia [49–51], has also been shown to influence sleep–wake state discrepancy [20, 52–55]. Chan et al. [18] found that the behavioral components of CBT-I alone (i.e. stimulus control, sleep restriction, and sleep hygiene and relaxation) reduced SOL discrepancy in older adults compared to those in the self-monitoring control group. The cognitive components of CBT-I may also impact sleep as they reduce dysfunctional beliefs and attitudes about sleep (DBAS) [56–59], which is related to improvements in sleep–wake state discrepancy [60]. Although monitoring of self-reported sleep is an integral part of CBT-I, objective sleep measurements are not routinely employed in CBT-I. Conventional CBT-I also does not include a component of supporting individuals with insomnia in understanding sleep-state perception.

The consumer market is seeing increasing varieties of wearable devices that objectively record sleep. Additionally, an increasing number of individuals with insomnia are presenting to sleep services with personally recorded sleep history, with the expectation that clinicians can understand and utilize their data [61–64]. Most sleep wearables utilize actigraphy (e.g. Fitbit), and some models are found to be comparable to research-grade altigraphs when detecting sleep/wake measures [65, 66]. A small number of head-worn wearables (e.g. Dreem) record electroencephalogram (EEG) signals and calculate sleep stages [67]. Although these devices sometimes provide consumers with feedback about sleep duration and quality (e.g. brief sleep variable descriptions, calculated sleep scores with descriptions), most do not provide detailed guidance about how to interpret the data in relation to users’ own sleep experiences. Some devices provide comparisons of the user’s data with those in the general population, which in some cases might cause a perpetuation of sleep-related anxiety that leads to a perfectionistic sleep quest (i.e. orthosomnia) [68]. This guidance for meaningful interpretation of data are especially important for individuals with insomnia, where sleep discrepancy might be high, and the aforementioned literature shows that different sleep feedback may result in different symptom outcomes.

Therefore, this randomized controlled study utilizes two conditions: one condition provides feedback on wearable measured sleep variables and discusses sleep–wake state discrepancy, and the other (control) condition provides information about sleep hygiene. This study aims to: (1) Examine whether providing individuals with insomnia feedback about sleep using sleep wearable devices, along with support for interpretation of sleep–wake state discrepancy improves symptoms of insomnia as the primary outcome. It is hypothesized that providing monitoring and feedback will result in a significantly greater reduction in insomnia symptom severity from baseline to post-intervention compared to control. Group differences in the changes of sleep and mental health domains (e.g. symptoms of depression, anxiety, and sleep-related daytime functioning) are also explored given their potential associations with both sleep perception and insomnia symptoms [4, 69–73], and (2) Explore potential mechanisms of change, including sleep–wake state discrepancy (operationalized as the differences between self-report and wearable measured sleep variables), dysfunctional beliefs and attitudes about sleep, presleep arousal, and aspects of sleep architecture (e.g. increased percentage spent in non-rapid eye movement [NREM] and rapid eye movement [REM], reduction of NREM and REM fragmentation, and increase slow wave density and amplitude).

## Methods

This is a two-arm, parallel-group, single-blind, and superiority randomized study. The study is prospectively registered here https://anzctr.org.au/, ACTRN12619001636145. This protocol paper follows recommendations from the SPIRIT-PRO Extension (Standard Protocol Items: Recommendations for Interventional Trials—Patient-Reported Outcomes) guidelines [74] and the template for intervention description and replication checklist and guide [75].

### Participants

Participants will be 90 individuals with significant insomnia symptoms. Inclusion criteria will be: (1) age ≥18 years, (2) able to communicate in English, (3) have regular access to the internet, smartphone, and email, and (4) Score ≥10 on the insomnia severity index (ISI) [76]. Exclusion criteria will be: (1) undertaking current fixed night shift work between 12 am and 5 am or current rotating work schedules that require night shift during the study period, (2) significant symptoms of the following sleep disorders based on duke structures clinical interview for sleep disorders (DSISD) [77]: sleep apnea (loud snoring, or observed gasping or pauses in breathing, or previously diagnosed with an apnea hypopnea index >15 but not/inadequately treated), periodic limb movement disorder (previously diagnosed with an arousal index >15), restless legs syndrome (occurring 3 times per week with a duration of at least 1 month), irregular and non-24 hours circadian rhythm sleep–wake disorders (advanced if habitual bedtime is earlier than 8 pm and habitual wake time earlier than 4 am, occasional deviation allowed; delayed if habitual bedtime later than 3 am and habitual wake time later than 11am, occasional deviation allowed), (3) at risk determined by individuals who select either moderate or high on the suicidal ideation item on the baseline questionnaire, and (4) using sleep information on Fitbit or Dreem apps in the month prior to participation.

### Procedures

This study was initially designed to recruit individuals from the Monash University Healthy Sleep Clinic (MUHSC; Victoria, Australia) for a mixture of in-person (e.g. study orientation session) and remote (e.g. phone) contact. Due to the coronavirus (COVID-19) pandemic, contact with participants will be completely remote (e.g. videoconference) and we will supplement recruitment with individuals from the community.

#### Recruitment

This study is promoted as the “Novel Insomnia Treatment Experiment (NITE)” and the following community and online recruitment strategies will be utilized: (1) At MUHSC, we will send recruitment invitations to (1) current patients who report significant insomnia symptoms (score ≥10 on the ISI at the intake questionnaire or are referred by a sleep physician) and have consented to be contacted about future research opportunities, and (2) past patients who gave consent to be contacted about future research opportunities. (2) Study recruitment materials will be placed in the general community Australia-wide, these included relevant online forums, social media, sleep websites, podcasts, and flyers placed in appropriate public spaces (e.g. notice board).

#### Informed consent

The online participant information and consent form contain extensive details about the research project such as information regarding interventions, assessment, research procedures, potential risks, reimbursement, confidentiality, and freedom to withdraw from participation. Once interested, individuals will provide informed consent via a web-based survey form, and they will then be booked in for telephone screening call.

#### Telephone screening

The telephone screening will take about 30 minutes and will cover: DSISD [77], medication use, engagement with psychotherapy or sleep treatments, and a risk assessment. Individuals who meet exclusion criteria due to the presence of other sleep disorders will be encouraged to discuss their conditions with a health professional, with appropriate referrals being made whenever necessary. Individuals eligible for the study will be booked in for a study information session and are sent the baseline questionnaire.

#### Randomization and blinding

Eligible participants will be randomized using permuted block randomization generated in advance. Block designs with varying block sizes of 4, 6, and 8 are used. Random seeds are generated to assure allocation concealment and pre-guessing of the allocation sequence at the end of each block. Randomization is stratified by baseline ISI (≤14 and ≥15). The randomization scheme is generated and set up in research electronic data capture (REDCap) by a member of the research team (JFW) who is (1) not involved in the recruitment or delivery of the study intervention and (2) is not one of the principal investigators. REDCap (hosted at Monash University) [78] is a secure, web-based application designed to support data capture for research studies, with audit trails for tracking data manipulation and automated export procedures for data downloads. To randomize a participant, an authorized researcher will log into REDCap, enter eligibility and stratification data on the participant, and receive the group allocation. Follow-up study measures will be either self-completed or conducted by research staff who are blinded to the condition. Participants will not be blinded but asked to withhold group allocation from research staff during the follow-up study measures. The initial screening telephone interview will be carried out by a provisional psychologist (MAS) under the supervision of a clinical psychologist (BB).

#### Timing of assessments and intervention

**Figure 1.**
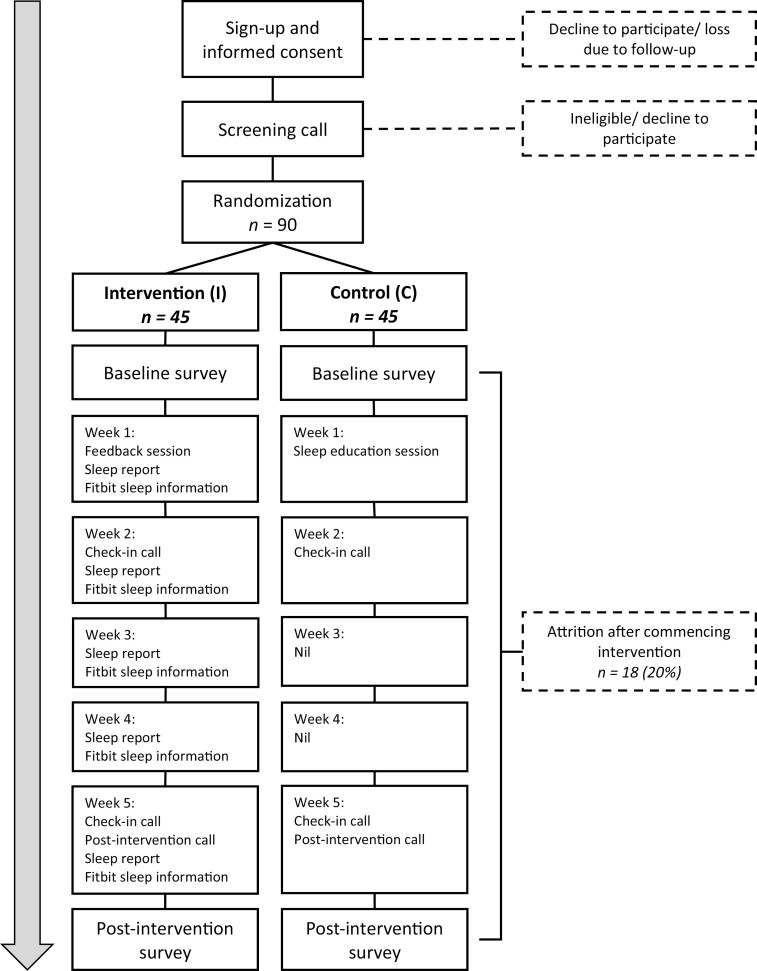
Novel inomnia treatment experiment (NITE) study flowchart.

Participants undertake online questionnaires administered via REDCap at two-time points: baseline (Baseline) and post intervention (Post). Figure 1 below contains a timeline of assessments and intervention material. All online questionnaires and telephone calls will be delivered by the research team in English.

All participants will attend two meetings (in-person or videoconferencing) with a researcher at baseline. The first meeting (~30 minutes) will cover study orientation and instruction regarding the research equipment. Following this, participants will complete the second meeting (~60 minutes for intervention, ~30 minutes for control; see details in the intervention condition section).

Participants also received four scheduled phone calls: (1) the aforementioned screening call, (2) two check-in calls 1 and 4 weeks after baseline (~10 minutes each) to encourage adherence and troubleshooting, and (3) a final telephone call will be made by a researcher blinded to group allocation at post-intervention to administer the DSISD Insomnia module (~10 minutes).

All telephone communication and videoconferencing sessions will be audio recorded with consent. Audio recordings are used for assessing reliability and intervention fidelity and are stored securely with password protection.

Participants will receive reminder emails to prompt questionnaire completion. Following the final assessment and the return of research equipment, participants will be sent up to a 70 AUD gift voucher as a token of appreciation for participating. The gift card amount is calculated based on the use of Fitbit and the completion of the sleep diaries. If participants use Fitbit for seven nights and complete the sleep diary for 5 nights per week, they will receive 10 AUD (up to 50 AUD for 5 weeks of participation). All participants will receive 20 AUD for completing online questionnaires.

#### Adverse events

Adverse effects will be monitored during each participant contact throughout the research project during the check-in phone calls, and via post-intervention questionnaire. Participants will also be instructed to contact the research team immediately if they experience unwanted adverse effects during participation so that intervention may be appropriately adapted (e.g. not using Dreem headband). Severe adverse events will be included in human research ethics reports.

#### Risk

A risk assessment will be undertaken during the initial screening telephone interview. It will assess suicidal/homicidal ideation, plan, history of injurious behavior, and engagement with mental health care. Participants deemed to be at high risk, defined as current suicidal/homicidal ideation with plan and/or history of harm will be excluded from the study and referred to relevant services for clinical care. Participants at medium risk, defined as experiencing current ideation to harm self/others but have no plan or previous history of harm, will be included if no other exclusion criteria are met; they are provided referral options for mental health care. Participants at low risk will be included if no other exclusion criteria are met.

#### Intervention conditions

Both interventions will include an individual session and two check-in calls. Interventions will be manualized to ensure consistency. A provisional psychologist undertaking doctoral training in clinical psychology (MAS) will be trained in the intervention, and competence in delivering the content will be assessed prior to study commencement by a clinical psychologist (BB). The provisional psychologist will receive regular supervision throughout the study. Participants in each group will be offered the content the other group received following the completion of the study.

#### Intervention condition

Participants in the intervention condition will receive the following components combined:

1 An individual session conducted by a provisional psychologist (MAS) that covers: (a) the differences between sleep variables measured using self-report and sleep measured using wearables, (b) the nature of sleep–wake state discrepancy and its relevance to insomnia, and (c) healthy interpretation of sleep–wake state discrepancy, through an assisted interpretation of participants' baseline sleep data (measured via Fitbit, Dreem, and self-report). Although the session draws upon strategies commonly used in CBT-I (e.g. addressing thoughts and beliefs related to sleep–wake state discrepancy), it will not include key components of CBT-I (e.g. stimulus control, sleep restriction).
2 Access to daily Fitbit sleep data through the Fitbit app, including TST, sleep start time, and wake time.
3 An illustrated weekly report (see Supplementary Material) that compares self-report (i.e. sleep diary) and objective (i.e. Fitbit and Dreem) sleep data. This report will be emailed to participants each week throughout participation and will include both daily and weekly summaries of: (a) TST (sleep diary and Fitbit), (b) SE (sleep diary and Fitbit), (c) SOL (sleep diary and Dreem), (d) wake after sleep onset (sleep diary and Dreem), and (e) sleep stages (Dreem). Data from two sources will be plotted together (e.g. sleep diary and Fitbit TST), and these figures will be accompanied by interpretations of discrepancies and information on how to use this information for managing concerns about sleep. These reports will be automatically generated weekly using an R-script that fetches data from Fitbit and Dreem servers via application programming interface (API).

#### Control condition

This control condition is designed to control for nonspecific factors such as receiving sleep information and time with a researcher. The control condition will be instructed to use the study devices (i.e. Fitbit and Dreem), however, they will not receive feedback about their objectively recorded sleep throughout their participation in this study. They will be able to utilize the Fitbit app for non-study related purposes (e.g. exercise, step tracking) but the sleep information tile will be hidden. They will receive their weekly sleep reports (see Supplementary Material) at the end of the study, following the completion of the final assessment. The control condition will receive a sleep education session conducted by the provisional psychologist (MAS) covering: (1) psychoeducation about sleep (e.g. information about what the functions of sleep, sleep cycles, sleep stages, the homeostatic sleep drive, and circadian rhythms), and (2) sleep hygiene (e.g. healthy sleep habits regarding light, body temperature, physical activity, noise, food, caffeine, alcohol, and bedding). Participants will not be instructed to alter any sleep-related behaviors.

### Measures

#### Daily sleep measures

The daily sleep diary that will be utilized in this study to assess self-reported sleep behaviors is adapted from the consensus sleep diary [79]. Participants will be asked to complete the sleep diary each day (~2 minutes per day; via computer or phone app) as soon as they have woken up, for the duration of the research project. The sleep diary collects information about experiences over the previous day and night (e.g. sleep timing, number of nighttime awakenings, naps, caffeine consumption, and medication for sleep). The consensus sleep diary has shown good clinical utility, usability, and ability to discriminate people with sleep dysfunction from normal sleepers [80].

Participants will be instructed to wear a Fitbit Alta HR (Fitbit Inc., 2017 release) on their non-dominant hands for the duration of the research project. The Fitbit Alta HR has been found to provide equivalent estimates to research-grade actigraphy for gross sleep parameters (e.g., total sleep time, sleep efficiency) in and outside of the laboratory [66, 81]. However, the Fitbit sleep staging is limited in accuracy compared to PSG [81–83], and is not used in this study.

Participants will be given an optional Dreem headband (Dreem Inc., version 2) to wear on their heads while sleeping for the duration of the research project. Compared to PSG, Dreem had high concordance for raw signal detection with 83.5% accuracy for overall scoring across five stages (i.e. all sleep stages and wake) and 74.0% accuracy with wake epochs alone (i.e. specificity) [84].

#### Structured interview

The DSISD [77] is a semi-structured clinical interview of sleep disorder, with the insomnia module amended to assess DSM-5-defined Insomnia Disorder. The DSISD will be administered in its entirety during the screening call, and the insomnia module will be repeated post-intervention by a researcher blinded to the participant’s group allocation. The DSISD has demonstrated good reliability and validity, with good interrater agreement for insomnia subtypes [77].

#### Questionnaires

Questionnaires include various self-report measures of sleep, mental health, fatigue, and beliefs about sleep among other factors. Table 1 describes when each measure will be administered.

**Table 1.** Timing of measurements. schedule of enrollment, interventions, and assessments

Timepoint	Items/ time	Enrollment	Allocation	Post-allocation		
				T_1_		T_2_
Enrollment						
Informed consent		X				
Phone call: Duke Structured Interview for Sleep Disorders & Assessment of risk	~30 min		X			X*
Allocation (randomized over phone)	~1 min		X			
Research groups						
Intervention Condition					**————**	
Control Condition					**————**	
Primary outcome						
Insomnia Severity Index	7			X		X
Secondary outcomes						
PROMIS Sleep Disturbance - SF	8			X		X
PROMIS Sleep-Related Impairment - SF	8			X		X
PROMIS Anxiety - SF	7			X		X
PROMIS Depression - SF	8			X		X
Fatigue Severity Scale	9			X		X
Epworth Sleepiness Scale	8			X		X
Quality of Life (AQoL-4D)	12			X		X
Presleep Arousal Scale	8			X		X
Dysfunctional Beliefs and Attitudes About Sleep	16			X		X
Glasgow Sleep Effort Scale	7			X		X
Other factors						
Demographics with mental and physical health history	~2 min			X		
Medication Use	~2 min			X		X
Credibility Expectancy Questionnaire	6			X		
COVID-19 Questionnaire	~2 min			X		
Study Feedback Scale (2 versions)	~2 min			X		

AQoL-4D, assessment quality of life 4D; PROMIS, patient reported outcome measurement; SF, short form; T1, baseline; T2, post-intervention; X, measure administered at that time point; X*, duke structured interview for sleep disorders insomnia module.

#### Primary outcome

The ISI [76], a 7-item self-report measure of insomnia symptom severity, will be the primary outcome. Items such as “difficulty staying asleep” and “difficulty falling asleep” are rated with the respondent reflecting on the past 2 weeks on a five-point Likert-type scale, ranging from 0 = none to 4 = very severe. Scores range from 0 to 28, with 0–7 = no clinical insomnia symptoms; 8–14 = sub-threshold insomnia; 15–21 = moderate clinical insomnia; and 22–29 = severe clinical insomnia [85]. Using 10 as a cutoff has demonstrated 86.1% sensitivity and 87.7% specificity for detecting clinically significant insomnia, with high internal consistency (Cronbach’s *α* = 0.90) in a community sample [86].

#### Secondary outcomes

1. Sleep quality measured using the Patient-Reported Outcome Measurement Information System (PROMIS) sleep disturbance short from [87]. It is an 8-item self-report measure assessing sleep disturbance. It is comparable psychometrically to the Pittsburgh sleep quality index [88], with a better ability to discriminate different levels of sleep disturbance [87, 89], and has good internal consistency (Cronbach’s *α* = 0.84) [90].
2. Sleep-related impairment measured using the PROMIS Sleep-Related Impairment Short Form [87]. It is an eight-item self-report measure assessing wake quality (e.g. daytime sleepiness, functioning), and it has been shown to have good internal consistency (Cronbach’s *α* = 0.84) [90].
3. Anxiety and Depression symptomology were measured using the PROMIS-anxiety Short Form and PROMIS Depression Short Form, respectively [91]. The PROMIS Anxiety (seven items) and PROMIS Depression (eight items) have both demonstrated good content validity and high internal consistency with Cronbach’s *α* = 0.95, and 0.93, respectively [91, 92].
4. Fatigue is measured using the Fatigue Severity Scale [93]. It is a 9-item self-report measure assessing the severity of fatigue during the past week in a variety of different situations (e.g. physical functioning). It shows excellent internal consistency and reliability (Cronbach’s *α* = 0.93), and stable test–retest reliability [94].
5. Trait sleepiness is measured using Epworth Sleepiness Scale [95]. It is an eight-item self-report scale assessing daytime sleepiness in adults, it has good test–retest stability after 5 months (*r* = 0.82) and high internal consistency (Cronbach’s *α* = 0.88) [96].
6. Health-related quality of life measured using the Assessment Quality of Life 4D [97]. It is a 12-item measure that assesses quality of life in four domains: independent living, relations, mental health, and the senses [97].
7. Presleep arousal is measured using the Presleep Arousal Scale [98]. It includes eight-item assessing cognitive arousal (e.g. being mentally active, alert) and eight-item assessing somatic arousal (e.g. heart racing, pounding) experienced at bedtime when attempting to sleep. The Presleep Arousal Scale shows satisfactory internal consistency and test–retest reliability in a range of settings, and validity has been demonstrated for populations with insomnia versus good sleeper controls [98].
8. Sleep-related beliefs are measured using the DBAS-16 [99]. The DBAS-16 is a 16-item self-report measure that assesses individuals' periods of sleep beliefs and attitudes in four domains (perceived consequences of insomnia, worry/helplessness about insomnia, sleep expectations, and medication) and allows for evaluation and change monitoring. DBAS-16 has adequate reliability, Cronbach’s *α* = 0.77 for clinical and *α* = 0.79 for research samples, and test–retest stability *r* = 8.83 [99].
9. Sleep effort is measured using the Glasgow Sleep Effort Scale [100]. It is a seven-item self-reported measure that assesses sleep effort and has been shown to be useful for clinical and change assessment. It reliably discriminates insomnia patients from good sleepers (cut off ≥2) and adequate internal consistency (Cronbach’s *α* = 0.77) [100].

#### Other measures

1. Demographic, mental/physical health history, and medication use using self-report at baseline (e.g. ethnicity, medical conditions, and family sleep history).
2. Participants’ perceived credibility and expectancy of treatment were measured using the Credibility Expectancy Questionnaire [101]. This is a brief six-item self-report measure of perceived “credibility” (i.e. how believable, convincing, and logical a treatment is to the patient), and “expectancy” (i.e. the improvements the patient believes will occur after treatment). The overall scale has high internal consistency (Cronbach’s *α* = 0.84 to 0.85) [101].
3. The impact of COVID-19 (Coronavirus) on project participation using the COVID-19 Questionnaire. Due to ongoing data collection during the COVID-19 pandemic, questions related to personal experiences with COVID-19 and potential impact on participation are asked to all participants who completed assessments after March 2020. This is intended to assist with interpreting findings from this study following completion.
4. Questions related to perceived helpfulness and feedback on each intervention component, as well as the sleep wearable devices.

#### Ethics considerations

Ethics approval is obtained from the Monash University Human Research Ethics Committee (MUHREC; 20856). All participants will provide informed consent for their participation. The consent will clarify that the decision of whether or not to participate in the study will not affect usual care and that participants are free to withdraw from the study at any time. Participants excluded due to current psychiatric conditions or severe comorbid sleep disorders will be encouraged to speak to their doctor about their mental health and medical needs. Any changes to the study protocol will be reported to and approved by the MUHREC before being implemented and are updated on the prospective registration.

Each participant will be assigned identification. Participants’ identifiable information will only be stored in REDCap, which has role-based secure access, and researchers working on this project or staff at the MUHSC have different levels of access (e.g. full access, de-identifiable information only). Access can also be immediately revoked if required. No identifiable personal information will be stored outside of REDCap, participants are only identified by their numeric identification. Only researchers approved by the MUHREC and staff of the MUHSC can access REDCap to re-identify participants. Participant's personal information (e.g. name, phone number) will be flagged and removed by REDCap when being used for research and/or service evaluation purposes.

No later than 7 years after the final publication, we will de-identify the data by removing names, date of birth, addresses/contact details, and any information that may link the data to participants personally. These personally identifying data will be completely erased and destroyed. The de-identified database will be made publicly available through Monash Bridges (or comparable platform) to maximize the potential benefit to the scientific and research community. Additionally, each variable will be screened, and rare categories with <5 individuals will be collapsed prior to sharing. No data monitoring committee will be employed in the current study due to limited scale, nor will study auditing be undertaken.

#### Statistical and power analysis

All analyses will be conducted on an intention-to-treat basis. Missing data is expected in this study design as participation occurs over several weeks and will be addressed using multiple imputations through chained equations. Pooled results from the multiply imputed data will be the primary analyses. Analysis will be conducted in R.

To examine group differences in primary and secondary outcomes, each outcome will be entered into a separate multiple regression model. Each outcome at post-intervention will be the dependent variable, with the outcome at baseline included as a covariate. Treatment conditions will be dummy coded and entered as the focal predictor. Stratification factors will be dummy coded and included as covariates in all models.

Each outcome will be evaluated for assumptions. If an outcome demonstrates substantial skew or non-normality, transformations (e.g. log, square root) will be evaluated to see if they improve normality. If outliers and extreme values are present, sensitivity analyses will use quantile regression to estimate the medians rather than linear regression as quantile regression models are less sensitive to extreme values. Scatter plots will be used to evaluate the association between each outcome at baseline and post-intervention. If there is evidence of substantial non-linearity, a generalized additive model will be used with a smooth term on the baseline outcome to allow non-linear associations.

In all cases, effect sizes will be calculated to quantify the size of group differences, 95% confidence intervals calculated around estimates of treatment group differences, and within-group change scores summarized to quantify the average change over time in each group. Sensitivity analyses will be conducted using only participants with complete data on each outcome. To explore predictors of treatment response, potential predictors will be incorporated in regression analyses as predictors or moderators.

Assuming an attribution rate of 20%, randomizing 90 participants in total (45 per group) will give the study 80% power (two-tailed *a* = 0.05, regression analyses) to detect a medium effect size (*d* = 0.6) on the ISI as the primary outcome. The effect size estimation is based on a previous behavioral intervention experiment, also included the ISI as the outcome, and addressed the discrepancy in self-report versus objective sleep information in individuals with insomnia [45].

## Discussion

The increased use of sleep wearable devices by consumers presents unique opportunities and challenges. Most sleep wearable devices provide quantitative information about sleep but do not provide support for interpretation. Current insomnia interventions (e.g. CBT-I), on the other hand, do not routinely incorporate objectively recorded sleep information into treatment. By examining whether adding guided interpretation of sleep wearable recorded sleep information could reduce symptoms of insomnia, this project has the possibility to uncover new approaches to supplement the current treatment of insomnia. As sleep feedback and guidance tested in this study will be automated, they have the potential of being incorporated into the reporting of sleep information in consumer sleep wearables. This has the capability of promoting a balanced interpretation of sleep state and benefits sleep health in the general public.

## Dissemination

Results will be disseminated through peer-reviewed scientific publications and conferences. If findings are promising, opportunities to further disseminate will be sought out, such as by contacting media (including social media) outlets and healthcare professionals and organizations with the aim of potentially being implemented into current sleep treatment and accelerating further intervention development. Participants who expressed interest in receiving study results will be provided with a summary of findings following study completion, including any resulting publications that may arise. Researchers who have made significant contributions to the design, conduct, analysis, and reporting of the current study will be granted authorship for planned and unplanned publications based on study data.

## Supplementary Material

zpad012_suppl_Supplementary_Material

## Data Availability

De-identified data from this study will be shared on reasonable request to the corresponding authors.
