# Supplementary material for: Does changing perceptions of sleep by incorporating sleep wearables improve insomnia? Protocol for a randomized study (the Novel Insomnia Treatment Experiment)
Source: Sleep Adv. 2023 Feb 16;4(1):zpad012. doi: 10.1093/sleepadvances/zpad012 (PMC10108650; doi:10.1093/sleepadvances/zpad012)

**Does changing perceptions of sleep by incorporating sleep wearables improve insomnia? Protocol for a randomised study (the Novel Insomnia Treatment Experiment “NITE”)**

Marie-Antoinette Spina^1^, Thomas Andrillon^3,4^, Joshua F. Wiley^1^, Shantha M. W. Rajaratnam^1^, Bei Bei^1,2^

1. Turner Institute for Brain and Mental Health, School of Psychological Sciences, Faculty of Medicine, Nursing and Health Sciences, Monash University, Victoria, Australia;
2. Women’s Mental Health Service, Royal Women's Hospital, Victoria, Australia;
3. School of School of Philosophical, Historical, and International Studies, Centre for Consciousness and Contemplative Studies, Monash University, Melbourne 3168, Victoria, Australia;
4. Paris Brain Institute, Sorbonne Université, Inserm-CNRS, Paris, 75013, France.

## Correspondence to: Bei Bei, DPsych(Clinical), PhD, Turner Institute for Brain and Mental Health, School of Psychological Sciences, Faculty of Medicine, Nursing and Health Sciences, Monash University, 18 Innovation Walk, Clayton Campus, Victoria 3800, Australia. [bei.bei@monash.edu](mailto:bei.bei@monash.edu).

## Supplementary Material – Sample of Weekly Sleep Report


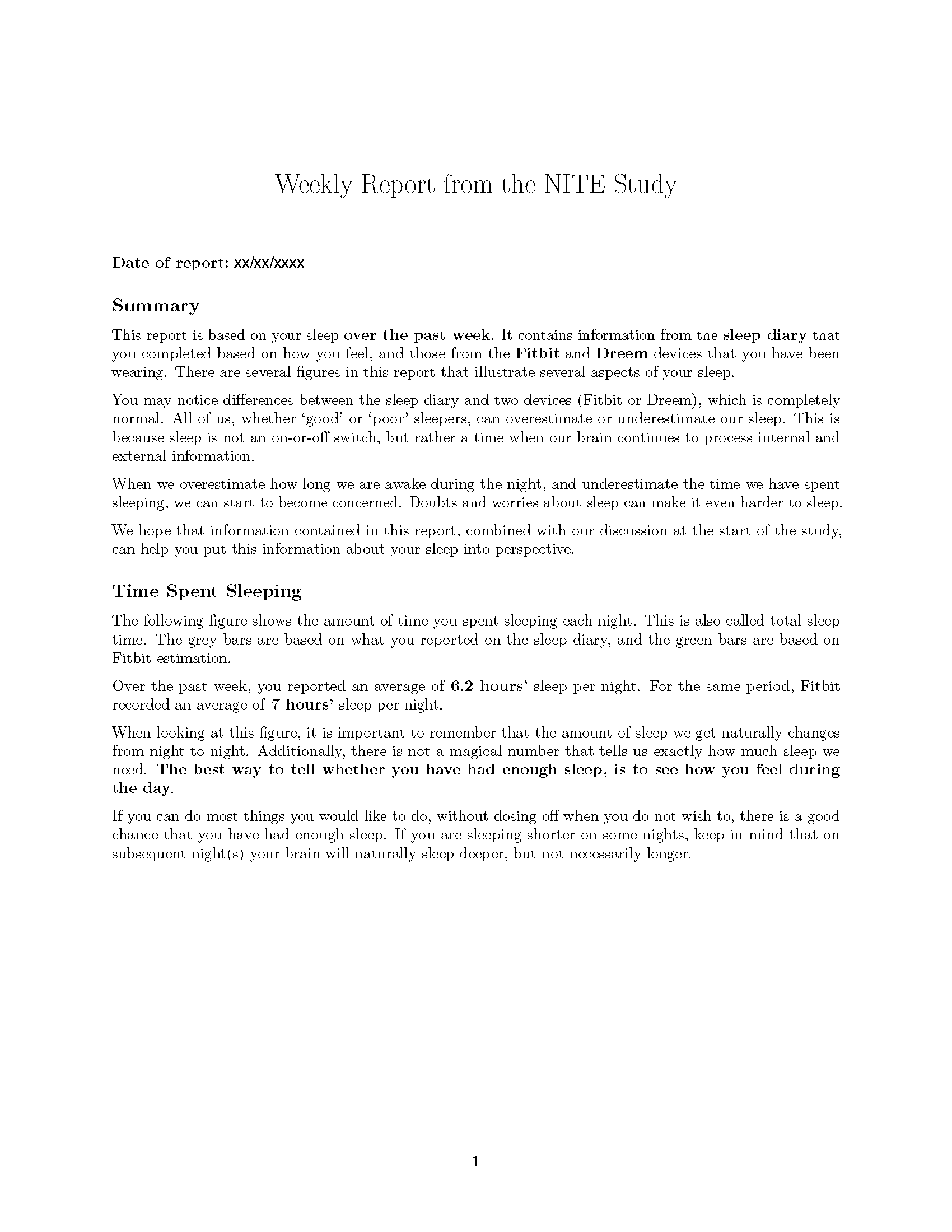


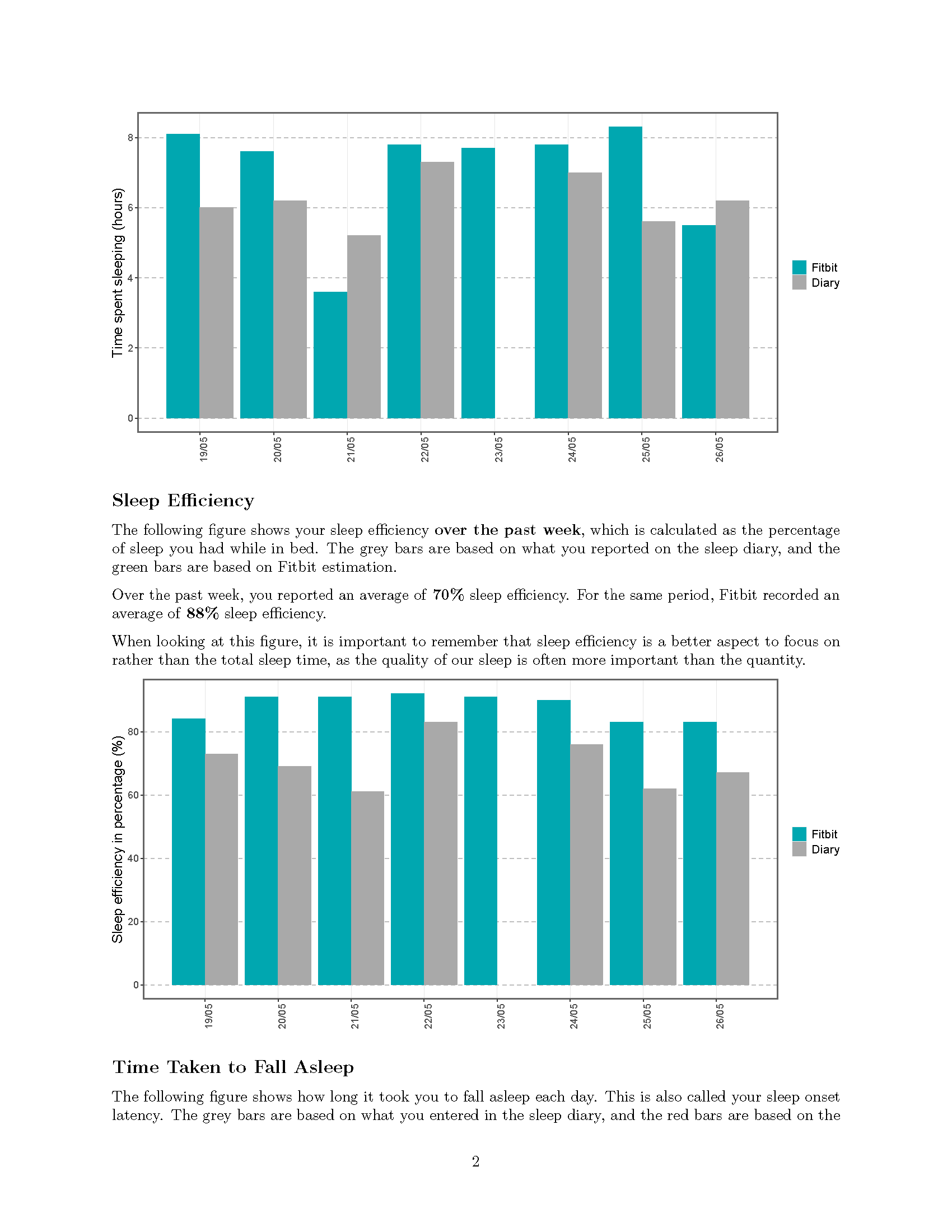


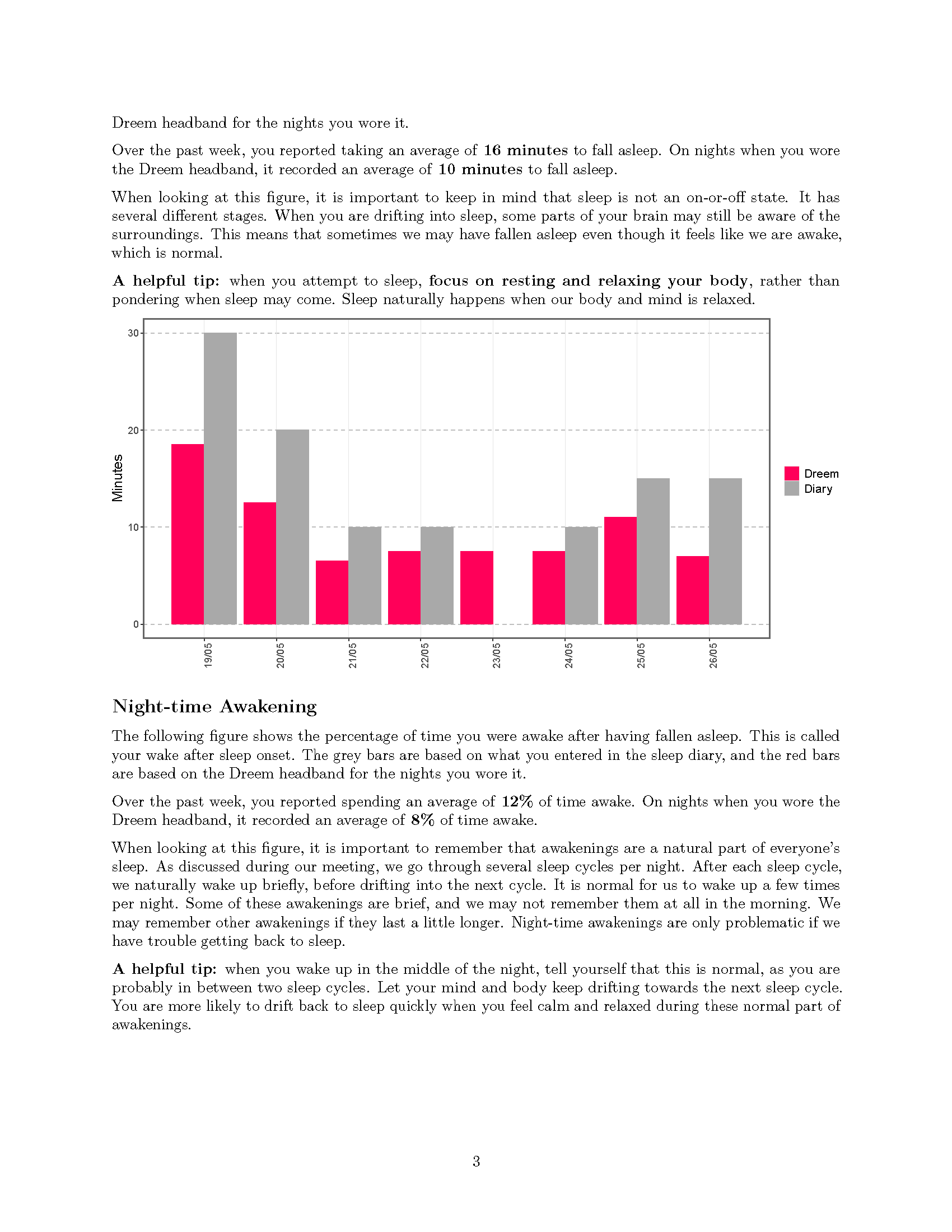


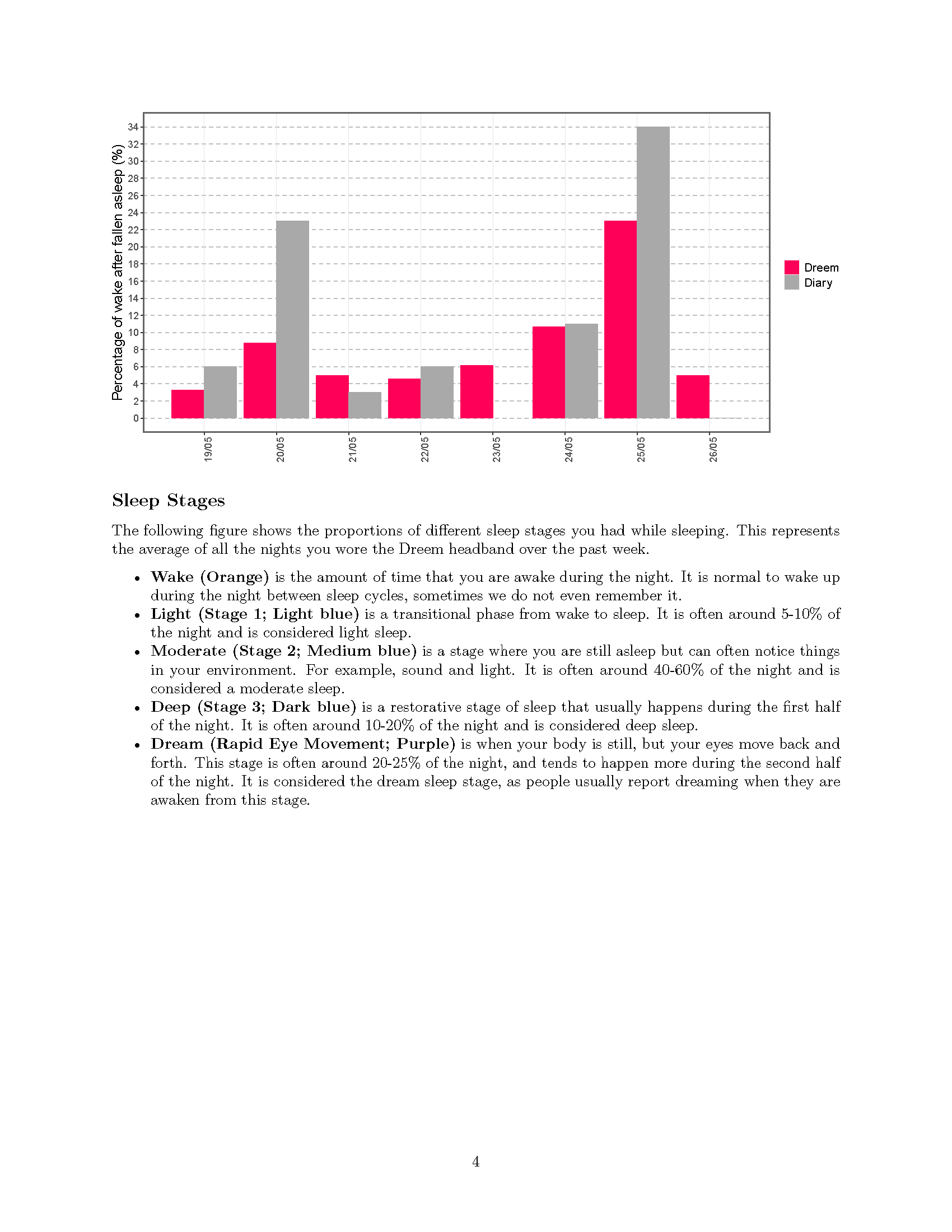


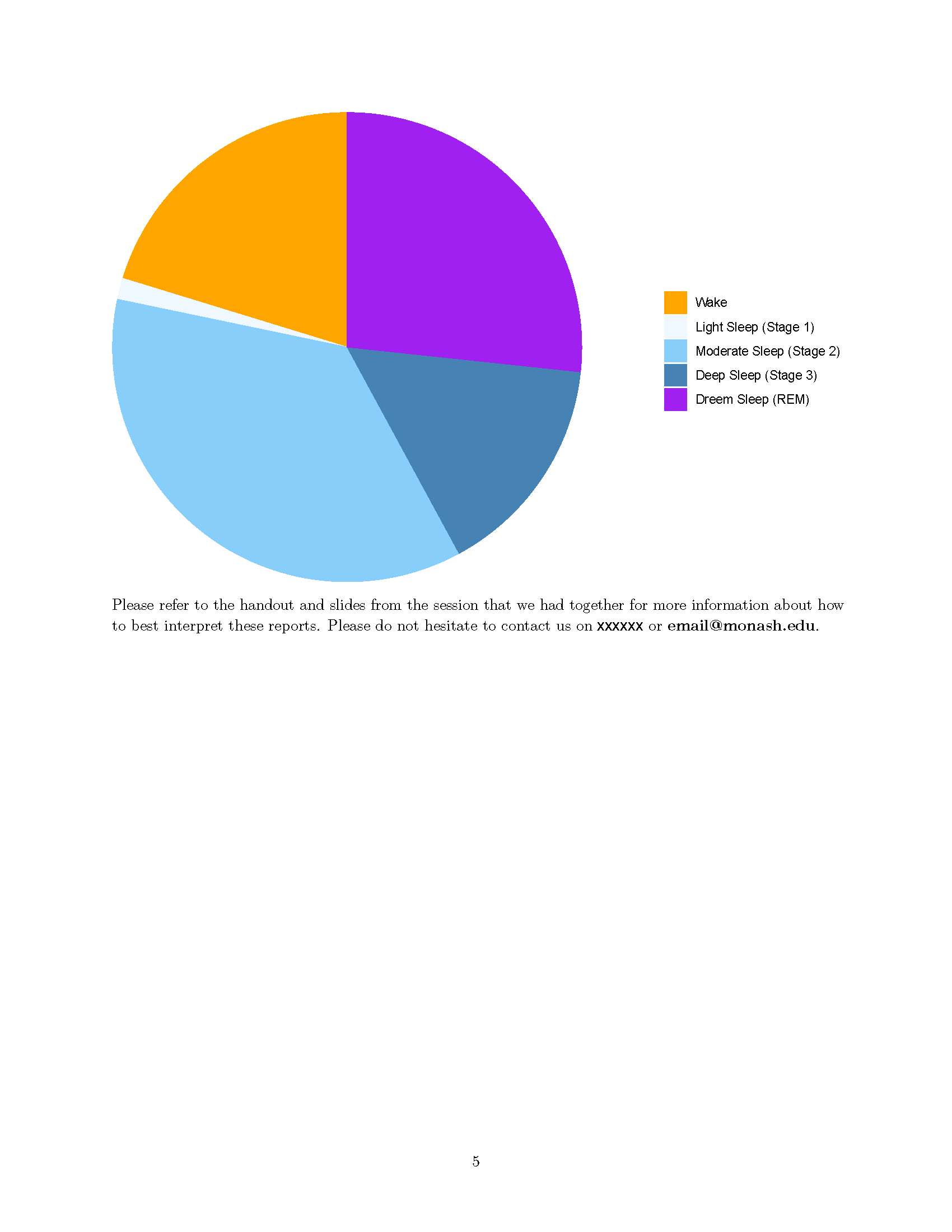


## Supplementary Material – SPIRIT Checklist
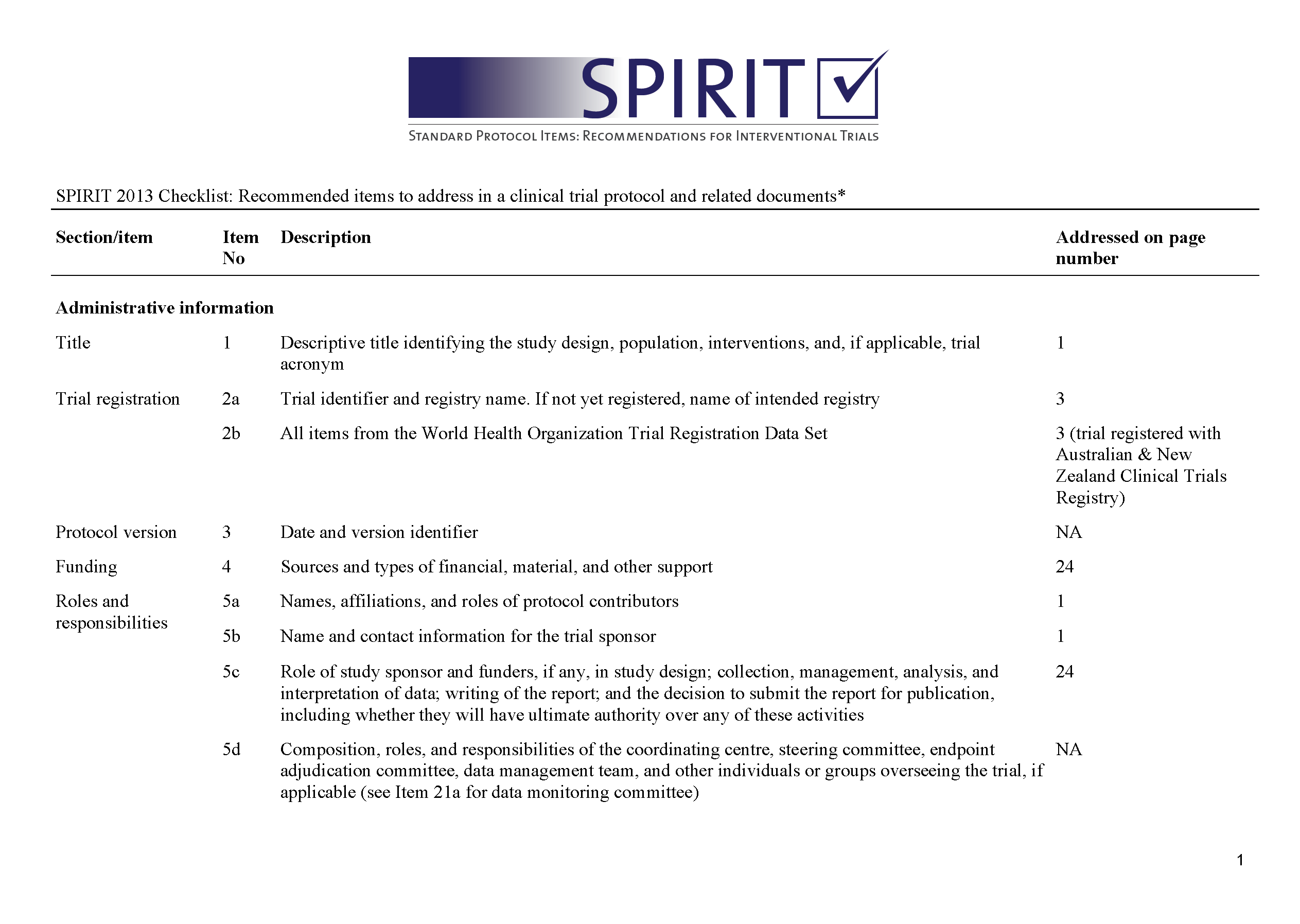


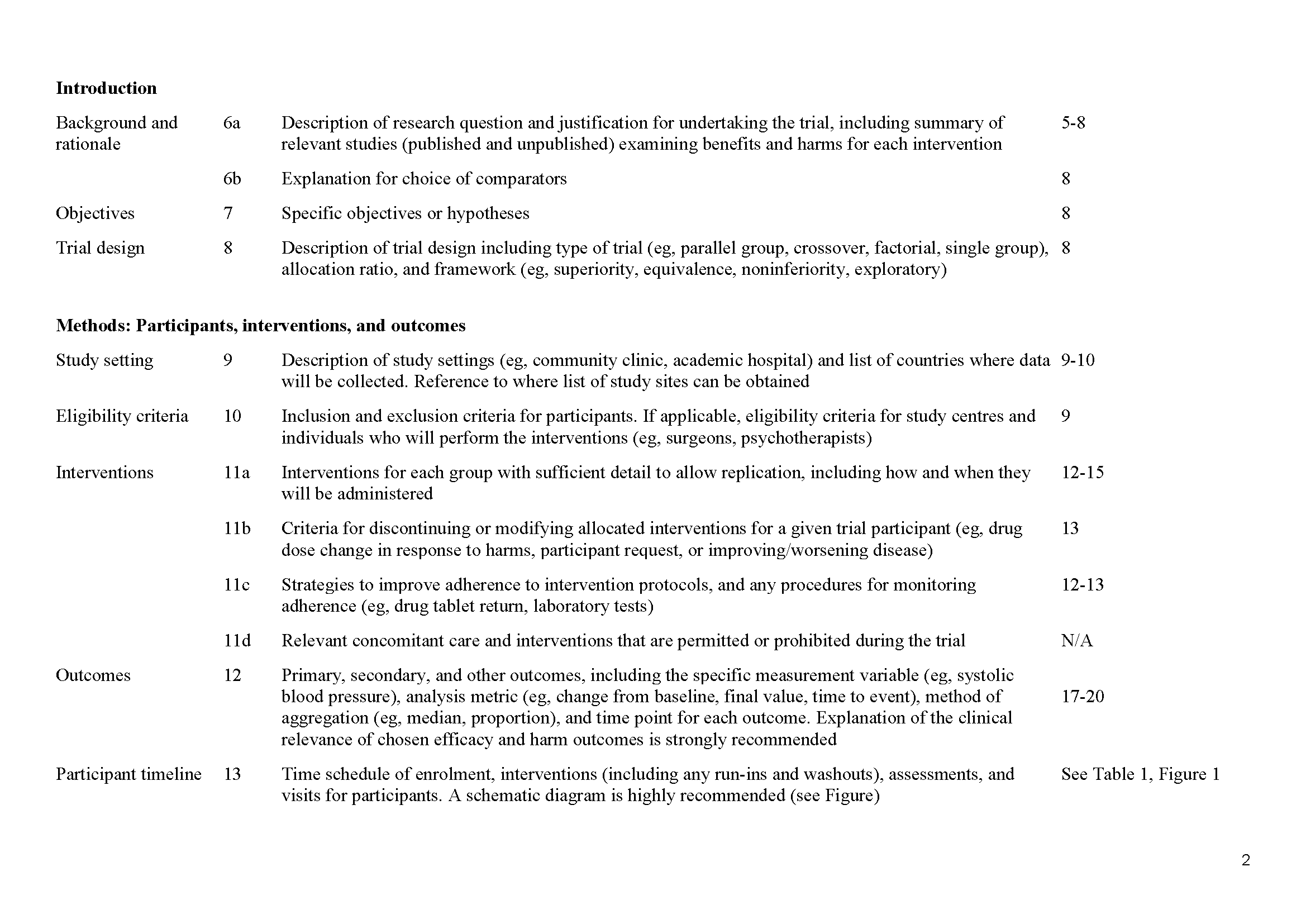


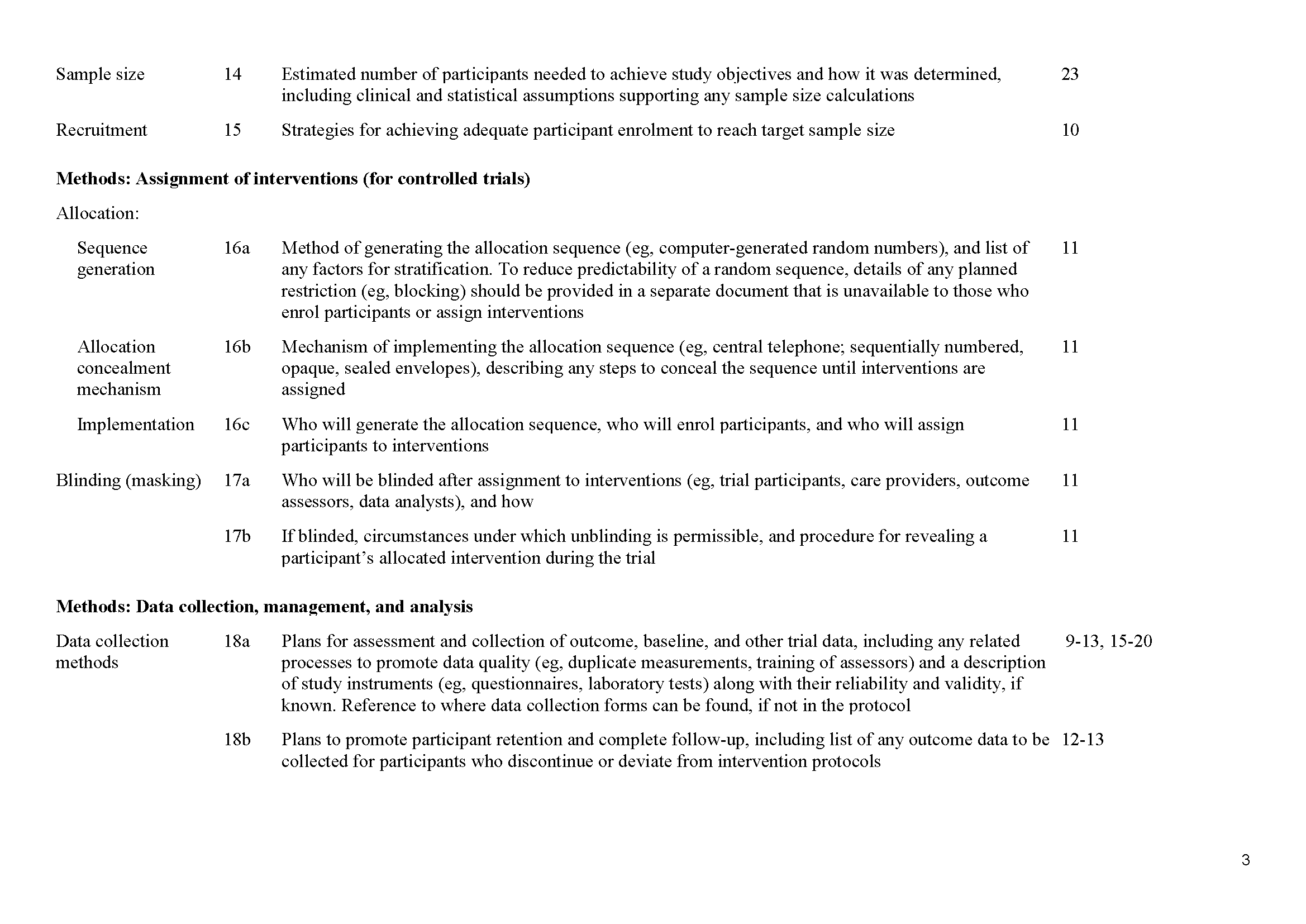


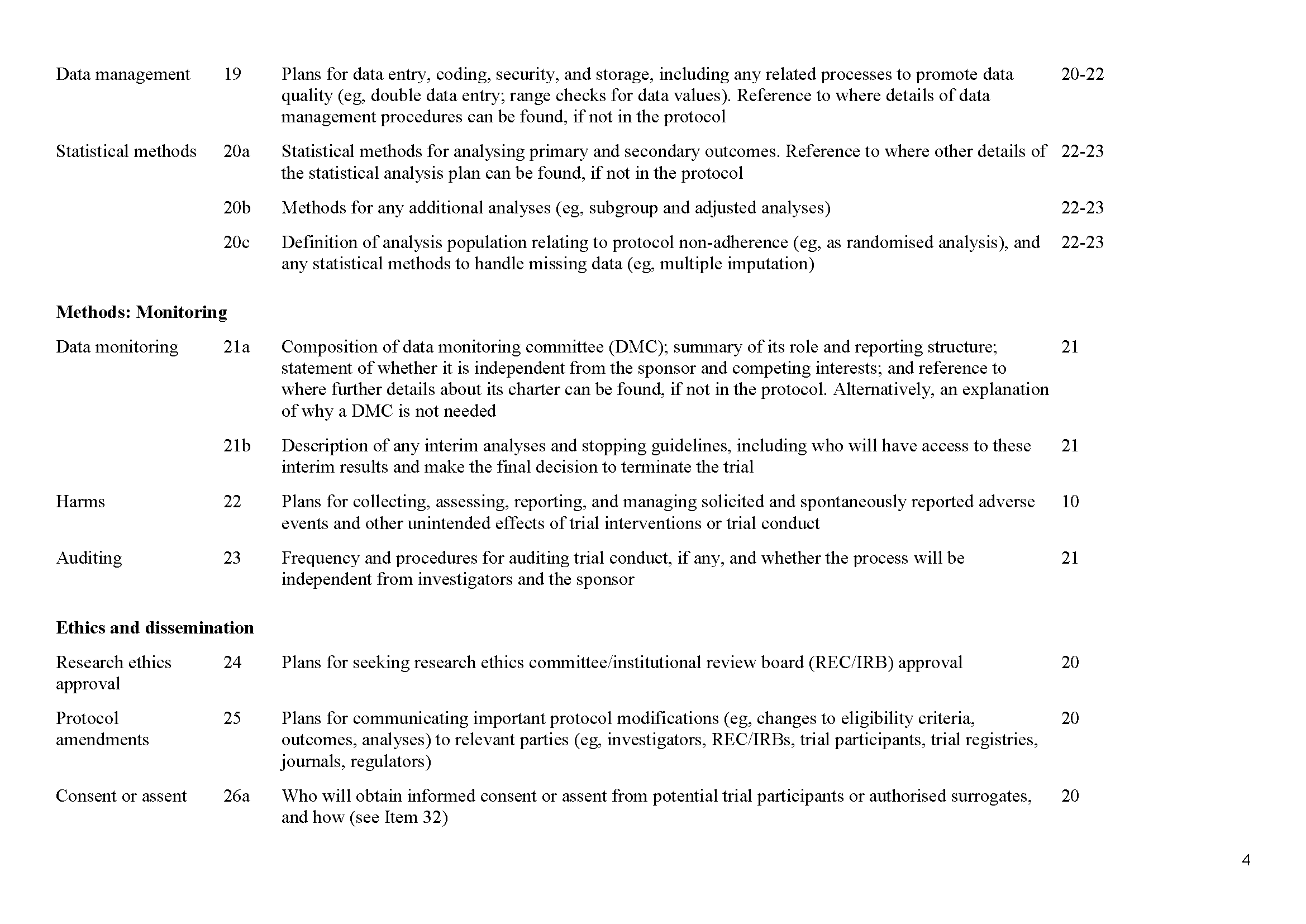


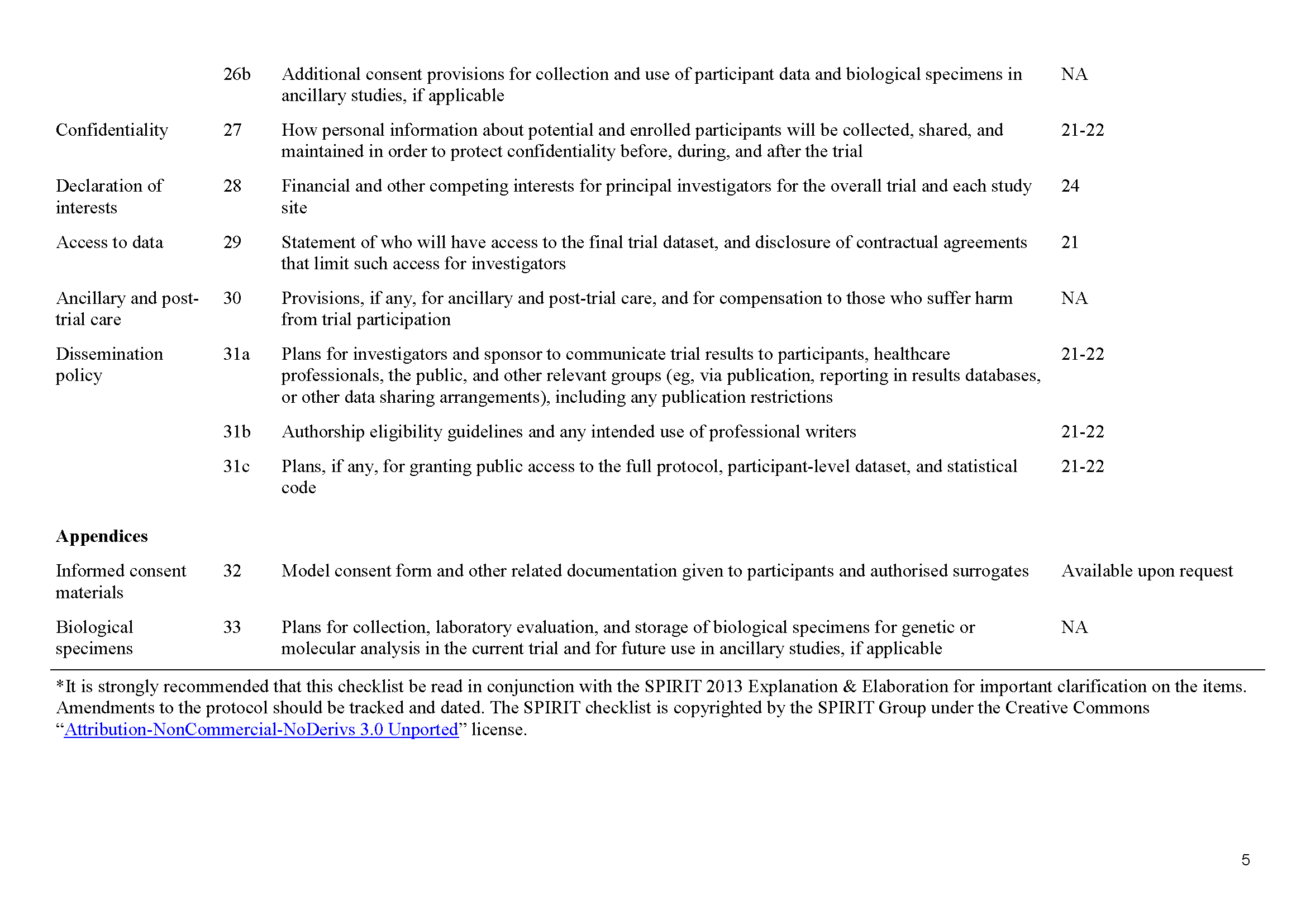


## Supplementary Material – TIDieR Checklist


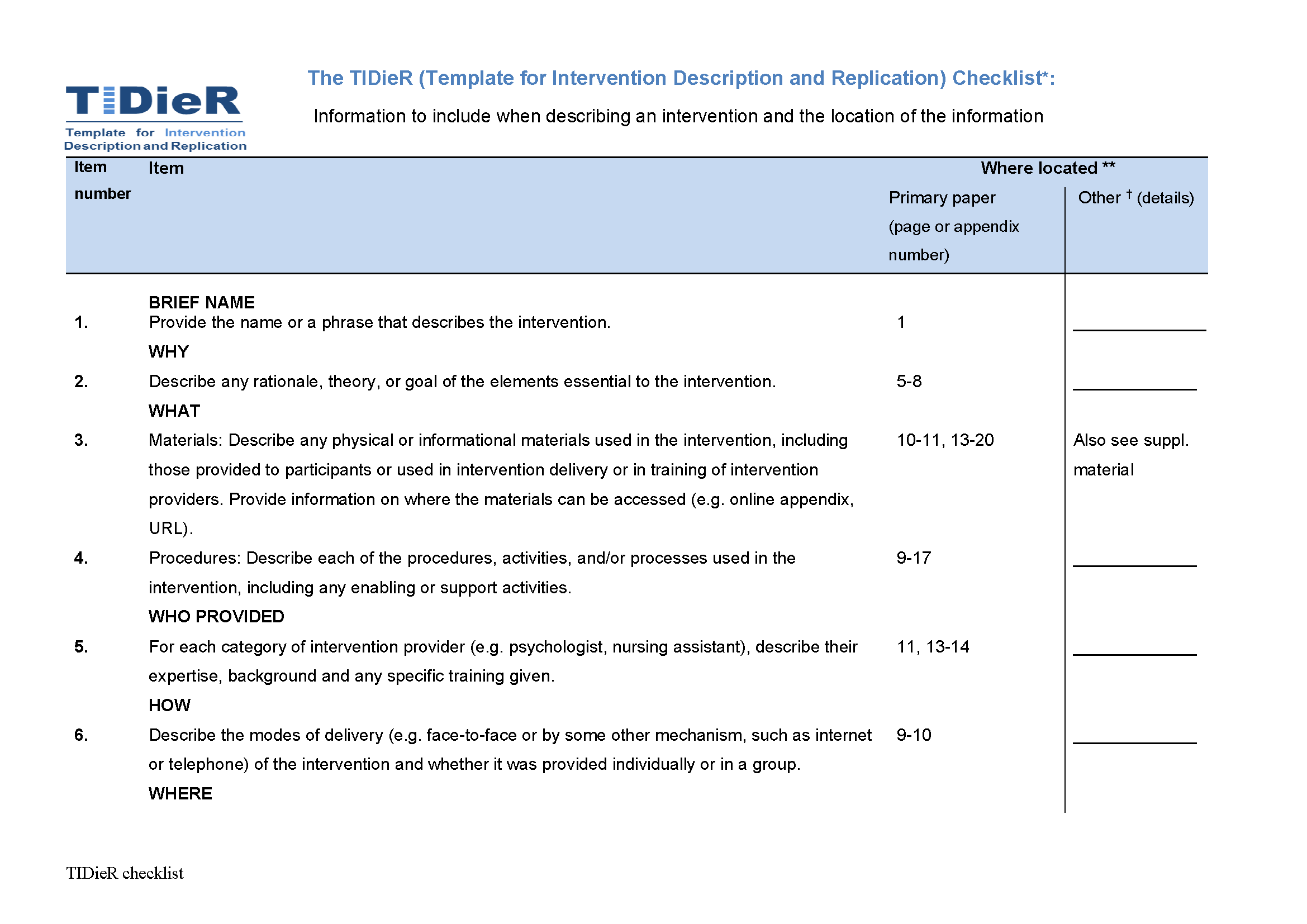


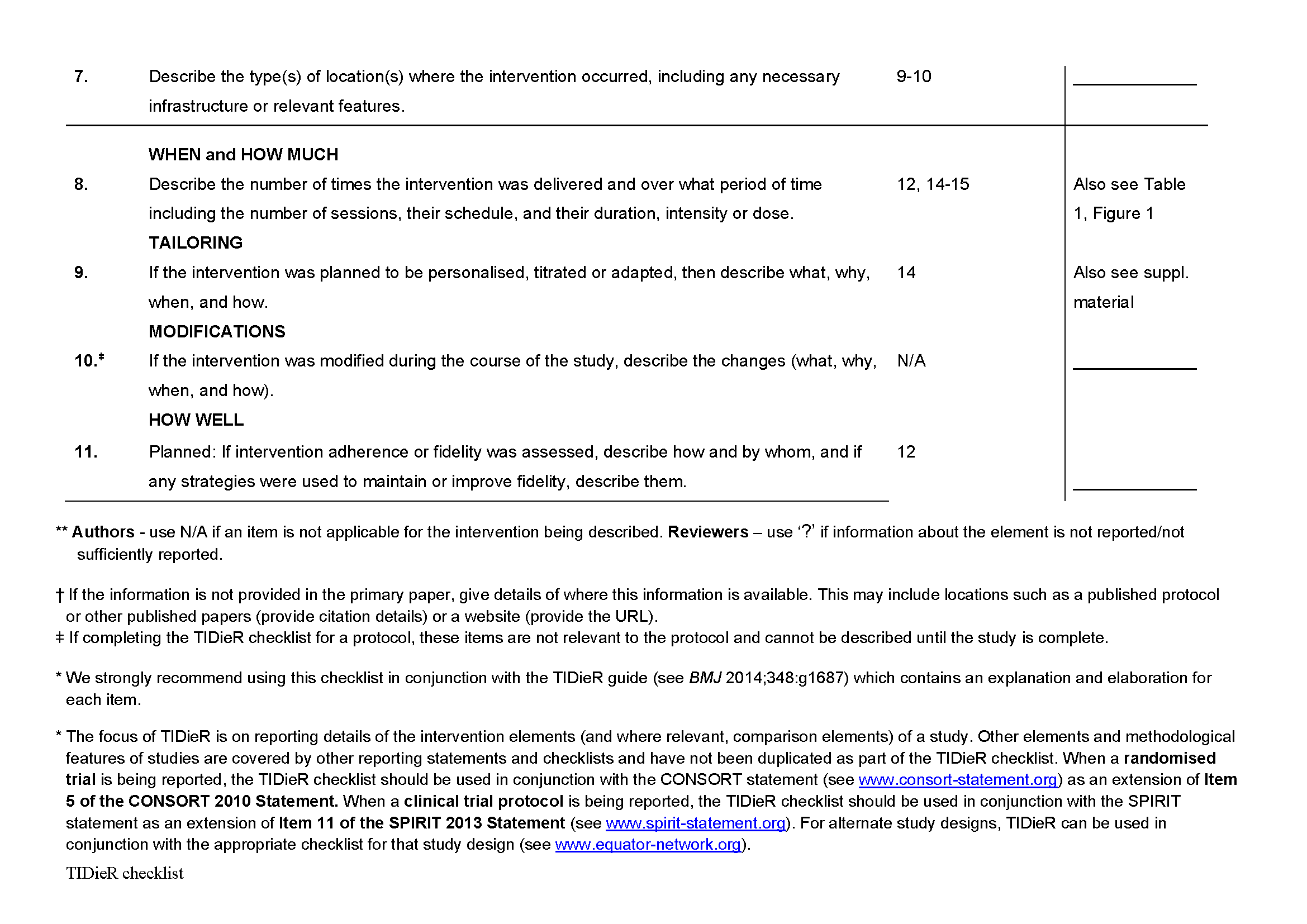

Supplement: zpad012_suppl_Supplementary_Material [file zpad012_suppl_supplementary_material.docx]
